# A simplified two-plasmid system for orthogonal control of mammalian gene expression using light-activated CRISPR effector

**DOI:** 10.1186/s12896-025-00994-2

**Published:** 2025-07-01

**Authors:** Shruthi S. Garimella, Shiaki A. Minami, Anusha N. Khanchandani, Justin C. Abad Santos, Susannah R. Schaffer, Priya S. Shah

**Affiliations:** 1 Department of Chemical Engineering, University of California, Davis, USA; 2 Department of Microbiology and Molecular Genetics, University of California, Davis, USA

**Keywords:** Optogenetics, CRISPR, CRY2-CIBN, Embryonic kidney cells, Myoblasts, Flow cytometry, Cotransfection

## Abstract

**Background:**

Optogenetic systems use light-responsive proteins to control gene expression, ion channels, protein localization, and signaling with the “flip of a switch”. One such tool is the *l*ight *a*ctivated *C*RISPR *e*ffector (LACE) system. Its ability to regulate gene expression in a tunable, reversible, and spatially resolved manner makes it attractive for many applications. However, LACE relies on delivery of four separate components on individual plasmids, which can limit its use. Here, we optimize LACE to reduce the number of plasmids needed to deliver all four components.

**Results:**

The two-plasmid LACE (2pLACE) system combines the four components of the original LACE system into two plasmids. Following construction, the behavior of 2pLACE was rigorously tested using optogenetic control of enhanced green fluorescent protein (eGFP) expression as a reporter. Using human HEK293T cells, we optimized the ratio of the two plasmids, measured activation as a function of light intensity, and determined the frequency of the light to activate the maximum fluorescence. Overall, the 2pLACE system showed a similar dynamic range, tunability, and activation kinetics as the original four plasmid LACE (4pLACE) system. Interestingly, 2pLACE also had less variability in activation signal compared to 4pLACE. We also demonstrate the optimal LACE system also depends on cell type. In mouse myoblast C2C12 cells, 2pLACE displayed less variability compared to 4pLACE, similar to HEK293T cells. However, 2pLACE also had a smaller dynamic range in C2C12 cells compared to 4pLACE.

**Conclusions:**

This simplified system for optogenetics will be more amenable to biotechnology applications where variability needs to be minimized. By optimizing the LACE system to use fewer plasmids, 2pLACE becomes a flexible tool in multiple research applications. However, the optimal system may depend on cell type and application.

**Supplementary Information:**

The online version contains supplementary material available at 10.1186/s12896-025-00994-2.

## Background

Inducible mammalian gene expression systems are valuable tools in research, biomedical, and biotechnology applications. They can control the expression of genes involved in processes such as stem cell differentiation, biosynthetic pathway optimization, and improved protein production in bioreactors [1–7]. Orthogonal modes to control gene expression can be especially useful depending on the application and can even be multiplexed. Inducible systems that use chemicals or temperatures to activate are easy to implement and reversible, but they can have off-target effects and lack precision [8–10]. Light-inducible or optogenetic systems function by using a specific wavelength of light to excite a light-activated protein engineered to control a biological process [11–14]. Optogenetic systems are uniquely suited for reversible and precise spatial control of gene expression because the light can easily be switched off and targeted to specific location via optical setups [15–18]. Owing to these many advantageous features, optogenetic systems are a promising technology for controlling scale-up of protein production or tissue engineering in biomanufacturing, specifically.

Several optogenetic systems have been developed recently using actuation by different wavelengths, including UV, blue, green, and red spectra [17, 19–23]. Red light systems may be of interest from an optical standpoint, since the lower energy light is less cytotoxic. In cells, red light also has lower absorption and scattering coefficients compared to blue light. Consequently, red light provides higher spatial control and deeper penetration into thicker tissue samples [23, 24]. The iLight and iLight2 systems take advantage of a conformational change in a red light-responsive protein to expose a DNA binding domain and drive gene expression [25–27]. The performance of these systems depends on the addition of exogenous biliverdin (BV) chromophore to reduce leaky gene expression in the dark. Other red/far-red systems, such as MagRed and REDLIP, display reduced leakiness without exogenous BV and leverage CRISPR-dCas9 for endogenous mammalian gene expression with minimal background expression [24, 28]. These systems have demonstrated red-light inducible gene expression in vivo to further illustrate its therapeutic potential. As red-light inducible systems improve, it becomes important to further develop additional inducible systems that operate in different wavelengths for multiplexing applications.

Blue-light systems are the most widely used optogenetic systems because of their higher chromophore efficiencies and the development of several light-responsive systems engineered to have improved performance [29, 30]. Light-Activated *C*RISPR *E*ffector (LACE) is one such system that takes advantage of protein dimerization following excitation by blue light. The light-responsive protein CRY2 is fused to viral protein 64 (VP64), a transcriptional activation domain derived from herpesvirus. CIBN is fused to deactivated-Cas9 (dCas9), which is targeted to a minimal CMV promoter or an endogenous promoter using an appropriate guide RNA (gRNA). CRY2 undergoes a conformational change when stimulated by blue light and dimerizes with CIBN. CRY2-CIBN dimerization brings VP64 to the promoter to activate gene expression. As a reversible system, gene expression turns off when light is removed, when CRY2 dissociates from CIBN [20, 31].

LACE is a powerful tool that enables modular optogenetics with flexibility to target exogenous and endogenous genes. Endogenous gene expression can be controlled if a suitable gRNA can be designed [20]. However, the LACE system requires delivery of four plasmids, which may limit how many cells receive all four components, especially in hard-to-transduce cell types. This limitation can increase variability in the cellular response, and increase the complexity of applying LACE to cell types relevant for biomedical and biotechnology applications [31]. Here, we improve upon the LACE system by decreasing the number of plasmids required and re-optimizing the ratio of the two combined plasmids. We compare two-plasmid LACE (2pLACE) to four-plasmid LACE (4pLACE) in human HEK293T cells and find that 2pLACE has similar dynamic range, tunability, and kinetics to 4pLACE with the added advantage of being more consistent. However, in mouse myoblast C2C12 cells we find that 2pLACE has decreased dynamic range compared to 4pLACE. Thus, 2pLACE provides improved outcomes for optogenetic applications, but these improvements may be cell line dependent.

## Results

### Design and plasmid ratio optimization of the 2pLACE system

**Fig. 1:**
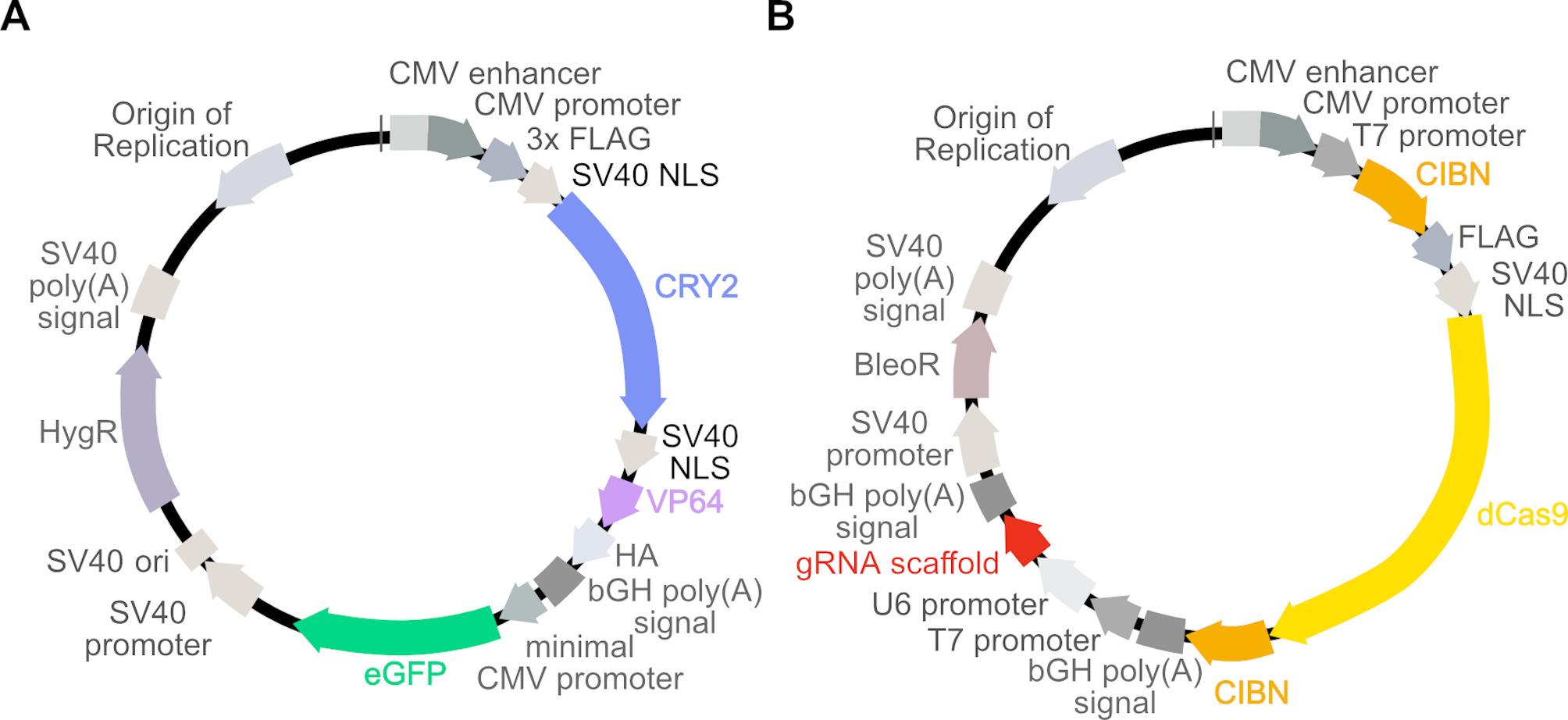
Schematic of 2pLACE design. (**A**) Expression cassettes for CRY2-VP64 and target gene (eGFP) with minimal CMV promoter and gRNA binding sites were combined into a single plasmid (CRY2-eGFP). (**B**) CIBN-dCas9-CIBN and gRNA expression cassettes were combined into a second plasmid (CIBN-gRNA). Each plasmid was designed to include an SV40 origin of replication (ori) for transient plasmid maintenance in cells expressing Large T-antigen. A distinct selection marker in each plasmid (Hygromycin and Bleocin) was included to enable stable cell line generation

We first set out to design a LACE system that would be easier to use and more efficient for various applications. The LACE system requires four different components to be delivered to cells. Originally, these four components were delivered on four separate plasmids by transient transfection [20, 31]. Consequently, the upper limit on LACE activation is dictated by the cells that receive sufficient amounts of all four plasmids. We therefore hypothesized that reducing the number of plasmids used to deliver LACE components would improve LACE behavior. We designed a system to reduce the number of plasmids from four to two by combining the CRY2-VP64 and minCMV-eGFP components and combining the CIBN-dCas9 and gRNA components (Fig. 1 and S1). These plasmids were synthesized commercially based on our design using a pCDNA3.1 backbone.

**Fig. 2:**
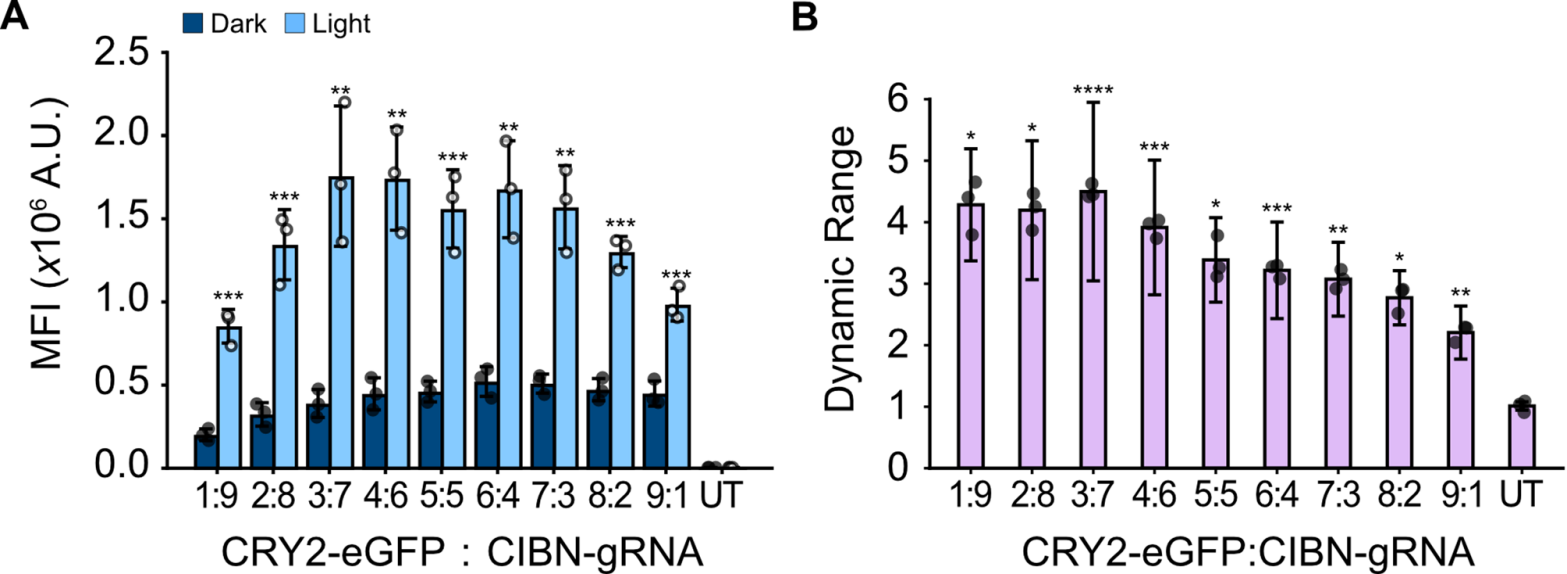
2pLACE plasmid ratio optimization for high absolute activation and dynamic range. (**A**) Mean fluorescence intensity (MFI) was measured for cells transfected with an increasing ratio of CRY2-eGFP: CIBN-gRNA plasmids followed by 24 h of incubation in light or dark. (**B**) Dynamic range for different plasmid ratios from (**A**) was calculated as MFI in light vs. MFI in dark. Plots represent the mean of three independent biological replicates and error bars represent standard deviation. UT (untransfected) is the negative control. MFI significance was calculated with a one-way t-test comparing the light and dark state of a single plasmid ratio. Dynamic range significance was calculated with a one-way ANOVA and Tamhane’s T2 post-hoc test to UT. P-values for comparison between other conditions are shown in Table S1. * *p* < 0.05, ** *p* < 0.01, *** *p* < 0.001, **** *p* < 0.0001, and ns is not significant

As an imbalance of the LACE components may influence leaky gene expression, maximal activation, and the dynamic range, we optimized the mass ratios of the two plasmids. Cells were kept in the dark or activated with pulsing blue light for 24 h, and eGFP reporter fluorescence was analyzed through flow cytometry. As the ratio of CRY2-eGFP to CIBN-gRNA plasmid was increased, the background expression of eGFP in the dark consistently increased. Expression of eGFP following activation also increased as a function of plasmid ratio, but peaks at a ratio of 3:7 and slowly decreased beyond that (Fig. 2A and S2). Dynamic range (ratio of light: dark signal) was significantly higher than dynamic range for untransfected cells for each plasmid ratio tested (Fig. 2B). Interestingly, the dynamic range started high but decreased as the CRY2-eGFP to CIBN-gRNA ratio increased beyond 6:4. The dynamic range for ratios 6:4 and beyond were significantly lower than dynamic range for the 3:7 ratio (Table S1). This suggests that there may be several suitable plasmid ratios. To balance high eGFP expression following light activation and high dynamic range, the ratio of 3:7 was used for all subsequent experiments.

### Characterization of transient 2pLACE activation dynamics

**Fig. 3:**
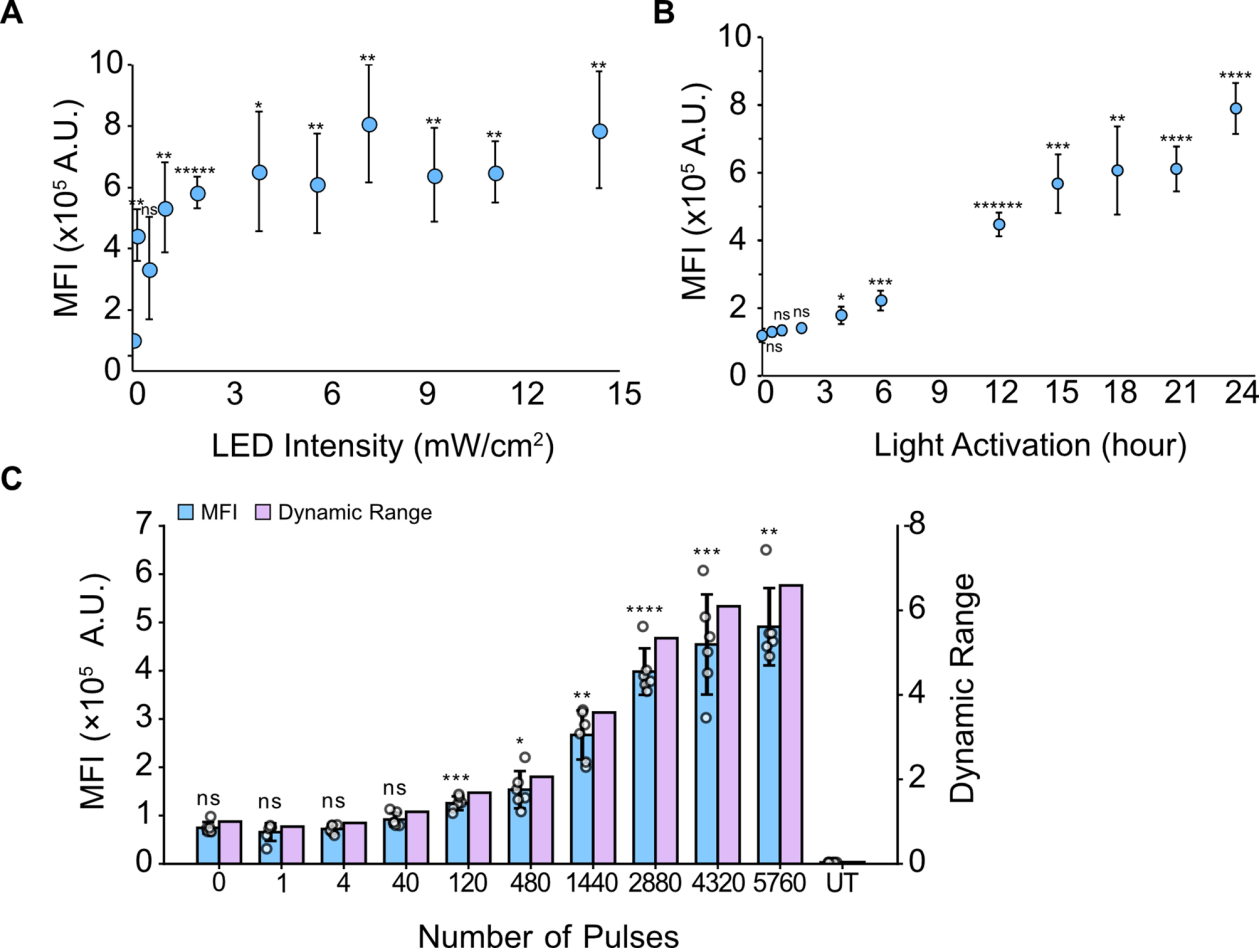
Characterization of 2pLACE activation dynamics. (**A**) eGFP MFI as a function of LED intensity. (**B**) eGFP MFI as a function of activation time. (**C**) eGFP MFI and dynamic range as a function of the number of LED pulses applied. UT (untransfected) is the negative control. Plots represent the mean of six technical replicates and error bars represent standard deviation. MFI significance was calculated with a one-way ANOVA and Tamhane’s T2 post-hoc test to the dark state condition with 0 h of light activation or 0 pulses. P-values for comparison between other conditions are shown in Table S2, Table S3, and Table S4, for each panel respectively. * *p* < 0.05, ** *p* < 0.01, *** *p* < 0.001, **** *p* < 0.0001, ***** *p* < 0.00001, and ns is not significant

Next, we measured the responses of the system to lighting conditions to assess tunability. We first tested the sensitivity of the system to LED intensity, where intensity was varied and eGFP fluorescence was measured 24 h post-activation. Experiments were performed using the optoPlate to enable high-throughput measurements in a 96-well format [32]. We found that the tunable range of eGFP expression occurred at intensities between 0 and 2 mW/cm^2^, with saturation behavior beyond those intensities (Fig. 3A). Significant activation occurred with light intensity as low as 0.12 mW/cm^2^, which was the lowest intensity tested (Figure S3). However, the activation was still quite variable for low intensities of light. Activation saturated around 2–3 mW/cm^2^ and maximum system activation occurred 7.26 mW/cm^2^. However, comparison between the conditions did not show any significant differences (Table S2). For subsequent experiments, we used an intensity of 9.23 mW/cm^2^. This corresponds to the intensity used to test the optimal mass ratio of 2pLACE (Fig. 2) as well as the intensity used in previous work [31]. Maintaining a similar intensity as the 4pLACE experiments also allows for direct comparison of system response.

We next measured activation kinetics. We activated cells transfected with 2pLACE for increasing amounts of time using the same pulse frequency. eGFP fluorescence was measured by flow cytometry. This time-course experiment showed that the system had an initial lag in expression before reaching significant but minimal expression as early as 4 h (Fig. 3B). 2pLACE’s activation continued to increase with prolonged activation time for significant expression (Table S3). The activation kinetics of 2pLACE confirmed that short exposure or activation times turns the system on, but the longer the system is exposed to light, the greater the protein can be expressed.

Our kinetics experiments suggest that prolonged exposure to light maximizes gene expression. However, blue light can be cytotoxic [33]. Moreover, some applications may benefit if the system can be activated with a short exposure to light [33–35]. We therefore evaluated the sensitivity of the system to the total number of pulses to determine if there was a critical threshold of pulses for efficient activation while still allowing time after activation for the protein to be expressed. Cells were transfected with 2pLACE and activated with blue light for the specified number of pulses and the same pulse frequency. eGFP fluorescence was measured at 24 h after pulsing was initiated (Fig. 3C). Significant activation occurred at 120 pulses, which corresponds to 0.5 h of activation time. Interestingly, this is lower than the activation time required for significant activation in our kinetics experiment (Fig. 3B) and suggests that there is a lag between CRY2 association with the promoter and the accumulation of a detectable amount of protein in this system. However, even though significant activation was observed for 0.5 h of pulsing, the reporter signal only reached substantial values starting at 1440 pulses, corresponding to 6 h of activation time (Table S4). Absolute activation and dynamic range continued to increase to 2880 pulses, which corresponds to 12 h of activation time. Thus, 2pLACE can activate gene expression with as little as 0.5 h of activation; however there are benefits to prolonged activation.

### Comparison of 2pLACE activation to 4pLACE

**Fig. 4:**
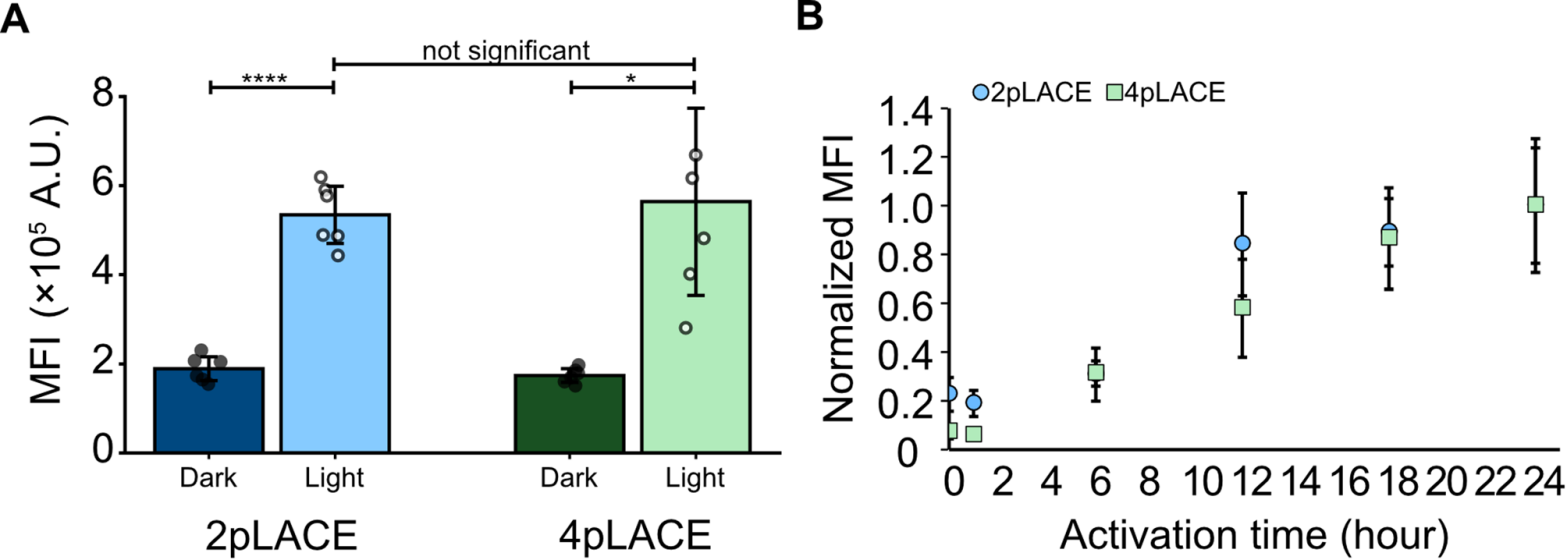
Comparison of 2pLACE and 4pLACE activation kinetics. (**A**) eGFP MFI was measured for cells transfected with 2pLACE and 4pLACE 24 h post activation with and without blue light. (**B**) Activation behavior of 2pLACE and 4pLACE over 24 h of activation. Data is normalized to the maximum activation of cells. Plots represent the mean of six technical replicates and error bars represent standard deviation. Significance was calculated with a one-way ANOVA and Tamhane’s T2 post-hoc test. * *p* < 0.05, **** *p* < 0.0001

By reducing the number of plasmids needed, the 2pLACE system offers more ease of use compared to 4pLACE. To this point, we directly compared the light activation characteristics of both systems. We measured eGFP fluorescence by flow cytometry with and without 24 h of light activation to understand the maximum expression of both systems (Fig. 4). 2pLACE achieves a similar magnitude of expression as 4pLACE, and notably with less variability (Fig. 4A). While there is no significant difference between the on states of the two systems, the consistency of 2pLACE is an improvement if the system is to be applied. We also compared activation kinetics of 2pLACE and 4pLACE over a 24 h period. Both systems activated with similar kinetic behavior (Fig. 4B). The matching activation profiles indicate the minimal system does not alter the dynamics of the LACE components. Thus, 2pLACE offers a similar activation profile to 4pLACE with improved consistency in activation.

**Fig. 5:**
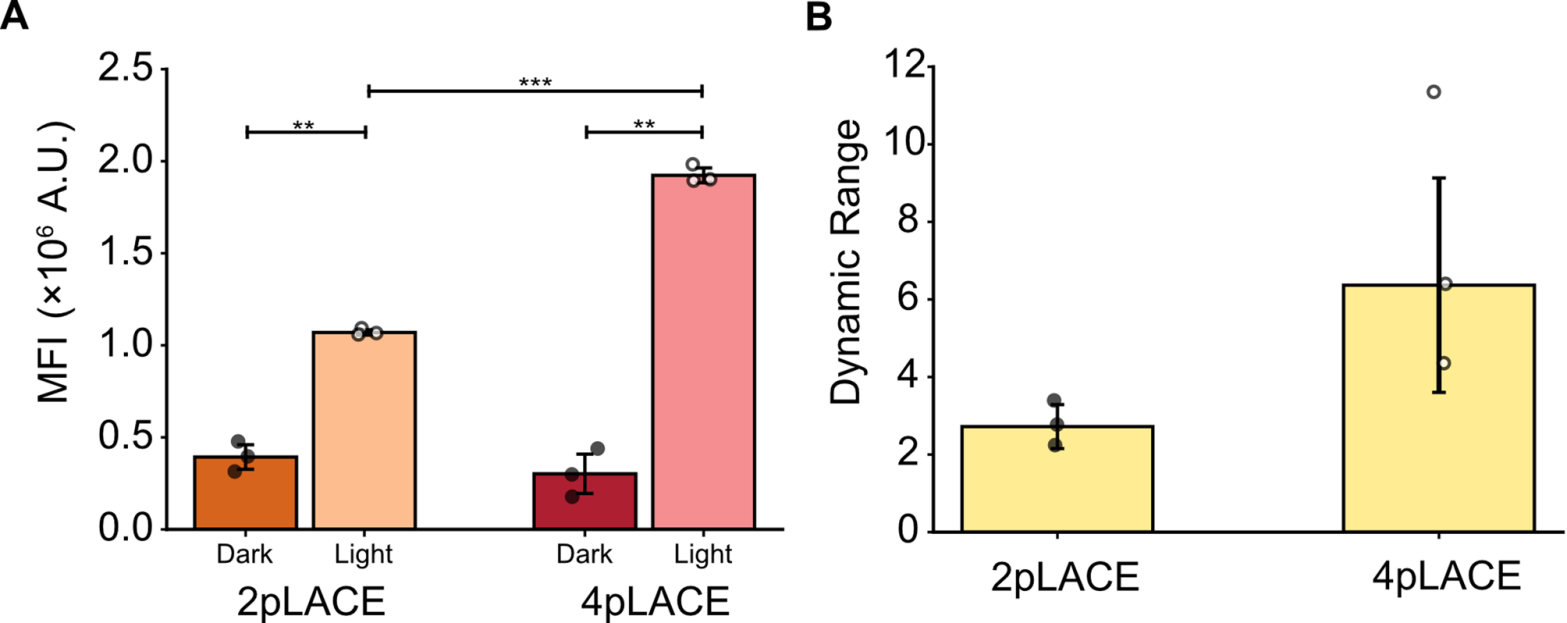
2pLACE and 4pLACE activation in C2C12 cells. (**A**) eGFP MFI was measured for cells transfected with 2pLACE and 4pLACE in C2C12 cells 24 h post activation with and without blue light. (**B**) Dynamic range of 2pLACE and 4pLACE in C2C12s after 24 h of activation. Plots represent the mean of three technical replicates and error bars represent standard deviation. Significance was calculated with a one-way ANOVA and Tamhane’s T2 post-hoc test. ** *p* < 0.01 and *** *p* < 0.001

We hypothesized that the reduction of plasmid numbers might improve activation efficiency, especially in hard-to-transfect cell lines in which delivering four plasmids may be a challenge. We therefore compared the activation of 2pLACE and 4pLACE in mouse myoblast C2C12 cells. This cell line is harder to transfect than HEK293Ts and a common model for muscle tissue engineering studies [36–39]. We used the same plasmid ratios and activation program we originally optimized for HEK293T cells. Interestingly, 4pLACE showed increased activation while having similar background expression compared to 2pLACE (Fig. 5A). This resulted in an overall higher dynamic range for 4pLACE, though this increase was more variable (Fig. 5B). Therefore, in C2C12 cells, 4pLACE has better activation than 2pLACE and overall system performance is cell-line dependent.

A major advantage of optogenetics over other inducible gene expression systems is spatial control. We therefore qualitatively tested 2pLACE spatial control of gene expression in C2C12 cells by masking cells from illumination using opaque tape. After 24 h of activation of 2pLACE and 4pLACE, we observed eGFP expression in the illuminated portion of cells but not the masked portion (Figure S4). Surprisingly, eGFP fluorescence from a constitutive CMV promoter was reduced in the light state on the illuminated side versus the masked side. No cytotoxicity was visible based on changes in cell morphology, and we hypothesize that reduced fluorescence was due to photobleaching of the eGFP from prolonged exposure to blue light. Thus, lower light intensities may be appropriate to avoid photobleaching when using eGFP as a reporter. Thus, the 2pLACE system is capable of spatial control of gene expression, similar to 4pLACE.

## Discussion

Optogenetic systems such as LACE are novel systems for inducible gene expression and have the potential to overcome limitations such as off-target effects, reversibility, cost, and lack of spatial control associated with other inducible systems. These advantages make LACE a powerful and flexible tool for a variety of applications, provided all four LACE components can be introduced to the target cells. However, the original LACE system was encoded on four different plasmids, limiting its potential use cases.

Here, we optimized the LACE system by reducing the number of plasmids needed to deliver the key components from four to two plasmids (Fig. 1). We varied plasmid stoichiometry and found that several plasmid ratios can provide similarly high dynamic range and a 3:7 plasmid ratio provides a balance for strong absolute activation and high dynamic range (Fig. 2). This reinforces findings by others that plasmid ratio optimization can be an important aspect of system performance for multi-component optogenetic systems [40]. As the LACE system relies on expression of four components for activation, the stoichiometric ratio of these components may influence performance. Indeed, the 4pLACE system, by nature of its modularity, may have performance benefits in cell types more sensitive to component ratios, as we observed with C2C12 cells. The LACE system also relies on the light-induced dimerization of CRY2 and CIBN, however, CRY2 oligomerizes upon blue-light excitation [41, 42]. Maintaining balance between the LACE components may also minimize the chance of CRY2 oligomerization such that CRY2 and CIBN interact to activate transcription.

The system can also be tuned using different blue light intensities, where significant activation occurs at 0.12 mW/cm^2^ and saturates after ~ 2 mW/cm^2^ (Fig. 3A). A kinetics time-course showed significant activation by 4 h but improving magnitude of activation with increasing activation time (Fig. 3B). Interestingly, when a short period of activation was followed by a prolonged period for gene expression products to accumulate, significant expression was achieved after only 0.5 h of light activation (Fig. 3C). This suggests there is a delay between complex formation at the promoter and translation. These results also reiterate that activation occurs on short timescales, but prolonged activation results in higher gene expression.

Comparing 2pLACE and 4pLACE directly in commonly used HEK293T cells showed that the systems have similar absolute activation and dynamic range after a maximum 24 h of activation; however the 2pLACE system provided a more consistent signal (Fig. 4A). We speculate that the improved consistency of 2pLACE comes from reduced variability in cells receiving sufficient amounts of all four LACE components. Reducing the number of plasmids from four to two also does not alter the activation kinetics (Fig. 4B). We hypothesize that many of the metrics between 2pLACE and 4pLACE are similar because the components have not changed. However, the reduced variability and the ease of use give 2pLACE improved potential for various synthetic biology applications.

Optogenetic systems like 2pLACE are low-cost inducible systems ideal for use in low resource environments. The implementation of optogenetics in industrial applications can ease the high costs of serums and antibiotics that are common to improve yield in bioreactors and activate chemically-induced systems [35]. For processes that require temporal control of gene expression, optogenetics also avoid costly media changes. 2pLACE’s ease-of-use means it can be applied to hard-to-transfect and more biologically relevant cell lines, such as primary cells used in tissue engineering for regenerative medicine and cultivated meat [43]. Cultivated meat is a growing area of interest as a sustainable solution for improving food accessibility [36, 44]. However, cost is critical to the industry’s viability. Easier-to-use optogenetic tools like 2pLACE could help bend the cost curve for the cultivated meat industry. For example, muscle stem cells benefit from expression of proliferative proteins for scale up, but these same proteins prevent differentiation into muscle cells [36, 45]. Temporal control of proliferative gene expression by 2pLACE could be used to strategically promote proliferation during scale up. Differentiation could proceed unimpeded with a flip of the switch to turn off blue light activation of proliferative gene expression.

To evaluate 2pLACE in cells more relevant to biotechnology and biomedical applications, we tested if 2pLACE would lead to improved expression in harder-to-transfect mammalian cells. We focused on C2C12 mouse myoblasts, which are commonly used in muscle development and tissue engineering research for its ability to differentiate into myotubes [36, 37, 46, 47]. Overall, we found that LACE performance and activation is greatly dependent on cell lines when we measure eGFP expression after 24 h of light activation. The change in performance aligns with other investigations that test blue and red optogenetic systems in different mammalian cells including COS-7, HeLa, and CHO-K1 cells [28, 48, 49]. While two plasmids improved the consistency of activation in HEK293Ts, the reduced plasmid number does not guarantee better activation. With C2C12s, we still see the consistent behavior of 2pLACE activation. However, 4pLACE does have a larger dynamic range than 2pLACE. This may be due to the reduced modularity in 2pLACE. It also suggests that, at least under the conditions we tested, transfection of all four components of 4pLACE was not a limiting factor. Further experimentation, such as reoptimization of plasmid ratios in C2C12 cells, and testing 2pLACE in a broader variety of cell lines and cell types are needed to fully understand why 4pLACE outperforms 2pLACE in some scenarios.

By optimizing the number of plasmids needed, 2pLACE shows more consistent behavior that improves its applicability as a tool in some scenarios. Yet, to be broadly applicable, additional improvements are still needed. Applying 2pLACE in industrial biotechnology settings will require stable expression of components to ensure activation over longer timescales. The work presented here characterizes 2pLACE under transient transfection conditions and transgene expression and are not necessarily compatible with long timescale processes or appropriate for evaluating control of endogenous gene expression. Our previous efforts to create a stable expression system for LACE using lentiviral vectors were hampered due to leaky expression in the dark state, and an inability to efficiently select cells that received all four components [31]. To this effect, we designed the 2pLACE system to have antibiotic resistance genes (Hygromycin and Bleocin, Fig. 1) as distinct selection markers for each plasmid. Again, having fewer plasmids to encode all four components may have advantages since fewer selection markers are required for stable cell line engineering. Stable expression of 2pLACE and efficient optogenetic control of endogenous gene expression will be essential to test for any future applications.

Additional development and optimization of single component systems could also improve the translation of optogenetics into industrial applications. Recent single component blue light systems in mammalian cells include a pdDronpa-CRISPR system, EL222, and a VP16-fused TULIP system [49, 50]. The pdDronpa-CRISPR system improves upon the dynamic range of the original LACE system but relies on higher energy violet light for dissociation. EL222 suffered from significantly low maximal reporter expression compared to red and alternative blue light systems in multiple mammalian cell lines. The VP16-fused TULIP system also resulted in less maximal transient reporter expression in a single component system when compared to a dual component VP16-fused TULIP system. Single component red light inducible systems leverage red light’s longer wavelength and minimal scattering to penetrate further in tissue samples and function in vivo. Recently, the red-light system REDLIP demonstrated transcriptional saturation at low intensities (~ 1.5 mW/cm^2^), suggesting it may be similarly sensitive to LACE and other blue light systems. It also demonstrated minimal expression in UV, blue, green, and far-red light, supporting its potential in multiplexed gene targeting [28]. However, blue light systems still have some advantages, like historically rapid activation due to higher energy light. Expanding the optogenetic toolbox with easier to use and more reliable blue light systems, like 2pLACE, could improve the ability to investigate and engineer more complex biological pathways, especially when multiplexed with other optogenetic systems [26, 51–54].

Finally, the simplicity and versatility of optogenetic systems like 2pLACE make them attractive for many applications, but it will still require specialized setups. For large-scale industrial applications, specific photobioreactors would need to be designed or current bioreactors would need to be outfitted with LEDs. We previously showed that blue light can propagate through a bioreactor to activate cells, but scattering is a major limitation that can dictate the upper limit on reactor size [35]. Specialized reactor designs with LEDs embedded throughout the reactor could also overcome the limitations associated with light propagation, though that may be costly. Ultimately, specialized smaller-scale applications like just-in-time or personalized medicine biomanufacturing may be more fruitful applications of 2pLACE and similar systems.

## Conclusions

In conclusion, LACE is a reversible and tunable optogenetic tool that can be designed to target a specific gene of interest given an application. By reducing the number of plasmids needed to deliver all necessary components, 2pLACE becomes a flexible tool for synthetic biology and bioproduction applications. The use of two plasmids ensures consistent signal while maintaining reversibility and tunability of the LACE system in HEK293T cells. However, performance of 2pLACE was highly cell type dependent. Additionally, while the requirement of blue light can limit its applicability in certain applications, 2pLACE broadens the accessibility of inducible gene expression tools for low-resource environments and biologically relevant models.

## Methods

### Plasmids

The 2pLACE plasmids, pcDNA3.1-Cry-GFP-Hygro and pcDNA3.1-CibN-gRNA-Zeo, were commercially synthesized (Genscript). Full plasmid maps are included as supplementary files. 4pLACE plasmids, pcDNA3.1-CRY2FL-VP64, pcDNA3.1-CibN-dCas9-CibN, pGL3-Basic-8x-gRNA-eGFP, and gRNA-eGFP-Reporter, were gifted from Charles Gersbach (Addgene plasmids #60554, 60553, 60718, and 60719, respectively).

### Cell culture

HEK293T cells (ATCC-No. CRL-11268) were cultured in DMEM (Gibco) supplemented with 10% FBS (Gibco). C2C12 cells were a gift from Lucas Smith at the University of California, Davis and were cultured in DMEM supplemented with 10% FBS and 1% penicillin/streptomycin antibiotics (Gibco). C2C12 cells were seeded at 5000 cells/cm^2^ in T-75 flasks and subcultured every 1 to 2 days before reaching 80% confluence. Cells were cultured in a humidified incubator at 37 °C and 5% CO_2_. Cell density and viability were measured prior to cell plating using trypan blue and an automated cell counter (TC20, Bio-Rad). Cells were tested monthly for mycoplasma by MycoStrip (Invivogen) or PCR with the primers targeting 16 S rRNA (Table 1).

**Table 1:** Primer sequences for mycoplasma PCR

Primer	Sequence
Primer 1	5’- TGCACCATCTGTCACTCTGTTAACCTC − 3’
Primer 2	5’- GGAGCAAACAGGATTAGATACCCT-3’
Primer 3	5’- GGCGAATGGGTGAGTAACACG − 3’
Primer 4	5’- CGGATAACGCTTGCGACCTATG − 3’

### Transient transfection

Twenty-four hours before transfection, HEK293T cells were seeded in a 96 well #1.5 H glass bottom plate (Cellvis) at a density of 0.035 × 10^6^ cells per well. Each well was transfected with a total of 100 ng of DNA. For experiments in a 12 well plate (VWR), HEK293T cells were seeded at a density of 0.016 × 10^6^ cells per well, with each well transfected with a total of 1000 ng of DNA. HEK293T transfections used PolyJet DNA In Vitro Transfection Reagent (SignaGen) with a PolyJet: DNA volume to mass ratio of 3:1, according to manufacturer instructions.

C2C12 myoblasts were seeded in a black 96-well plastic bottom plate (VWR) at a density of 5.0 × 10^3^ cells per well twenty-four hours prior to transfection. Immediately before transfection, media was replaced with fresh, pre-warmed growth media. Each well was transfected with a total of 300 ng DNA with the same ratios used with HEK293T cells. C2C12 cell transfections used TransfeX (ATCC ACS-4005) with a TransfeX: DNA volume to mass ratio of 2:1, according to manufacturer instructions.

The plasmid ratios for 2pLACE were relative proportions. Plasmid ratios for 4pLACE were calculated based on mass and molecular weight of plasmids relative to gRNA-eGFP-Reporter (Table 2) [20].

**Table 2:** Relative mass ratios of the transiently transfected LACE system plasmids

Plasmid name	Relative mass proportions	Mass per 100 ng (ng)	System
pcDNA3.1-Cry-GFP-Hygro	3	30	2pLACE
pcDNA3.1-CibN-gRNA-Zeo	7	70	2pLACE
pcDNA3.1-CRY2FL-VP64	2.06	20.6	4pLACE
pcDNA3.1-CibN-dCas9-CibN	2.94	29.4	4pLACE
pGL3-Basic-8x-gRNA-eGFP	4	40	4pLACE
gRNA-eGFP-Reporter	1	10	4pLACE

Transfections used PolyJet DNA In Vitro Transfection Reagent (SignaGen) with a PolyJet: DNA volume to mass ratio of 3:1 according to manufacturer instructions.

### Light activation

Cells were activated with LEDs at 465 nm twenty-four hours post transfection. For the optimization of plasmid ratios, a breadboard with 8 mm LEDs at a pulse frequency of 0.067 Hz was used to light activate the cells in a 12-well plate. Dark state samples were wrapped in aluminum foil to ensure isolation from light. For all other experiments, light activation was performed using an optoPlate-96 [32]. LED intensities and pulse frequencies were regulated with an Arduino Micro microcontroller. For experiments performed in 96-well plates, samples in the dark were wrapped in aluminum foil to ensure isolation from light. LED intensities were measured using the 1931-C Newport optical power meter.

### Flow cytometry

Under red light, cells were trypsinized (0.05% Trypsin-EDTA, Gibco) and resuspended in cold DPBS (Gibco) with 1% FBS (FACS buffer). After centrifugation at 4 °C for 10 min at 1000 RPM, 80% of the supernatant volume was removed and pellets were resuspended in 250 µL cold FACS buffer. HEK293T samples were wrapped in aluminum foil to ensure isolation from light following preparation for flow cytometry. C2C12 myoblasts were similarly prepared, except every two wells were transferred into a single BD Falcon 5 mL test tubes (352054, Fisher) for each replicate. A 488 nm laser in a CytoFLEX S Flow Cytometer was used for all analytical flow cytometry experiments. A target value of 10,000 total events was reached for every sample before gating for live, single, fluorescing cells. Cells were gated at an intensity of 5 × 10^3^ on the FITC-GFP channel. This corresponded to ~ 0.5% of cells falling into this gate for an untransfected negative control.

### Fluorescent imaging

Fluorescent images were taken using a ZOE Fluorescent Cell Imager (BioRad) after 24 h of illumination, unless otherwise specified, using the green and brightfield channels.

For spatial control, C2C12 mouse myoblasts were similarly prepared in a black 96-well plastic bottom plate with black electric tape covering half of the well surface to block blue light illumination from underneath.

### Statistical analysis

Statistical analysis was done using a one-way ANOVA test with Tamhane’s T2 post-hoc test for its conservative analysis because it assumes unequal variances where each condition was compared to all other conditions. Python packages scipy.stats, f_oneway, and scikit_posthocs were used for the ANOVA and post-hoc test. For statistical analysis using a one-tailed t-test, each light state was compared to its dark state. Python package scipy.stats’ ttest_ind function was used for the one-tailed t-test. The one-tailed t-test was done assuming the mean of the dark state was less than the mean of the light state making it a right-tailed t-test.

For the biological replicates, standard deviation was calculated with error propagated:

1 $$\:{\sigma\:}_{f}=\left|f\right|\sqrt{{\left(\frac{{\sigma\:}_{A}}{A}\right)}^{2}+{\left(\frac{{\sigma\:}_{B}}{B}\right)}^{2}-\frac{2{\sigma\:}_{ab}}{AB}}$$.

*σ*_*f*_ is the standard deviation of the dynamic range, *f* is the dynamic range value, *A* is the MFI value for a light activated sample, *B* is the MFI value for the dark state sample, *σ*_*A*_ and *σ*_*B*_ are their standard deviations, respectively. *σ*_*AB*_ is the covariance of the light and dark conditions. We assumed that the two conditions are not correlated, therefore the covariance is 0.

## Electronic supplementary material

Below is the link to the electronic supplementary material.

Supplementary Material 1

## Data Availability

The processed datasets supporting the conclusions in this article are included within the article and Supplementary Information. All raw data used to generate plots and materials are available upon request.

## Abbreviations

LACE: Light activated CRISPR effector
CRISPR: Clustered Regularly Interspaced Short Palindromic Repeats
dCas9: Dead Cas9
gRNA: Guide RNA
eGFP: Enhanced green fluorescent protein
MFI: Mean fluorescence intensity
