# Supplementary material for: A simplified two-plasmid system for orthogonal control of mammalian gene expression using light-activated CRISPR effector
Source: BMC Biotechnol. 2025 Jul 1;25:58. doi: 10.1186/s12896-025-00994-2 (PMC12220775; doi:10.1186/s12896-025-00994-2)
Supplement: Supplementary file 1 — Supplementary Material 1 [file 12896_2025_994_MOESM1_ESM.docx]

***A simplified two-plasmid system for orthogonal control of mammalian gene expression using light-activated CRISPR effector***

Shruthi S. Garimella^a^, Shiaki A. Minami^a^, Anusha N. Khanchandani^a^, Justin Cruz Abad Santos^a^, Susannah R. Schaffer^a^, and Priya S. Shah^a,b^

^a^ Department of Chemical Engineering, University of California, Davis, USA

^b^ Department of Microbiology and Molecular Genetics , University of California, Davis, USA

University of California, Davis

**Supporting Information**


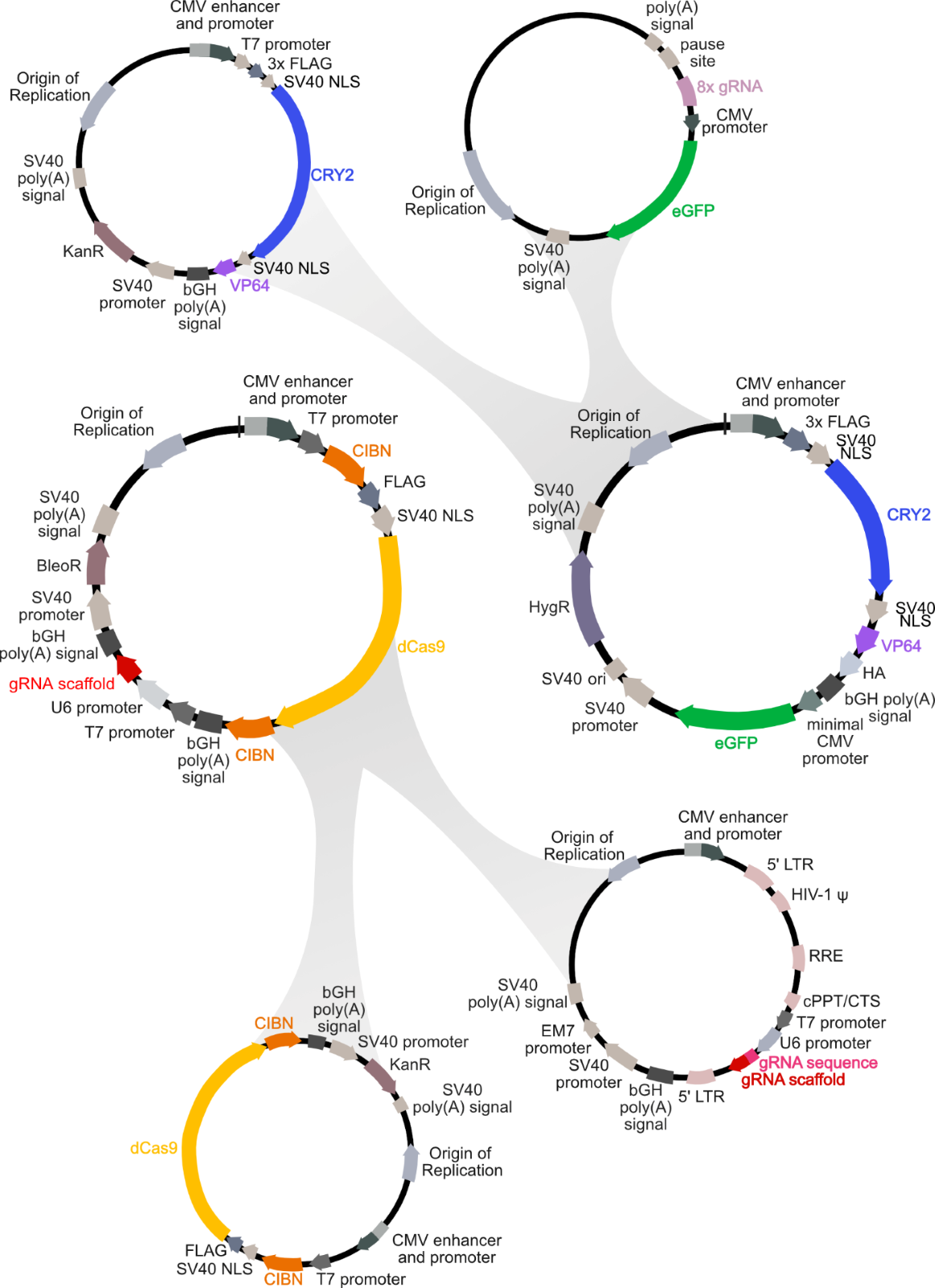


**Figure S1.** Schematic of plasmid design from the original four LACE plasmids (4pLACE) to the optimized two plasmids (2pLACE).


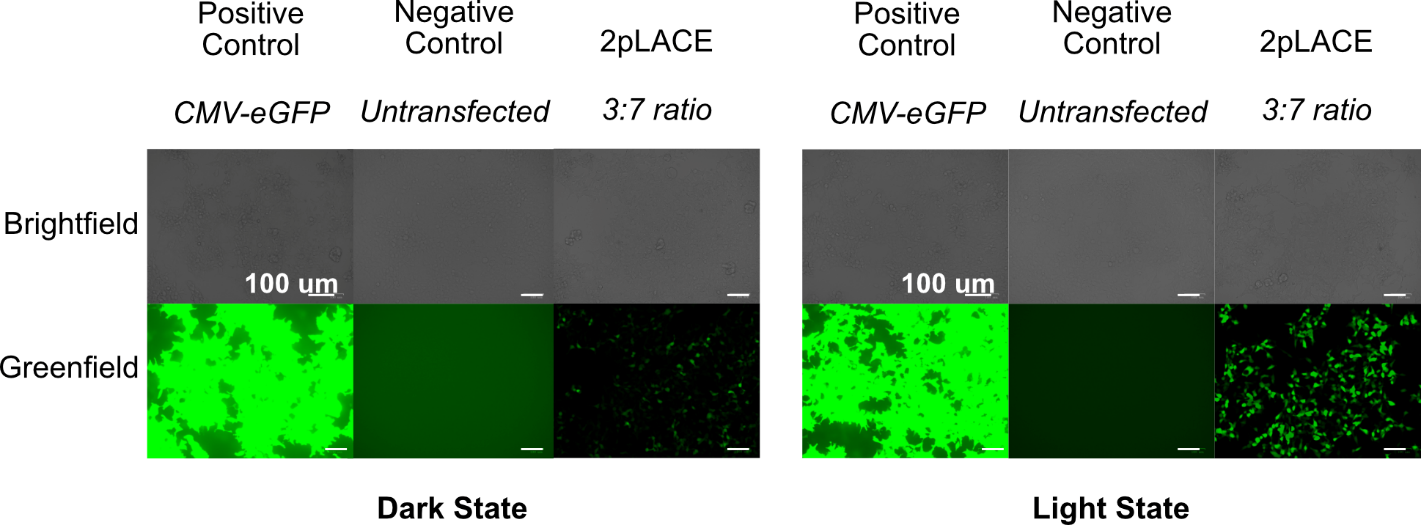


**Figure S2.** eGFP expression of the optimized plasmid ratio for 2pLACE with and without light activation. Images were taken 24 hours post activation.


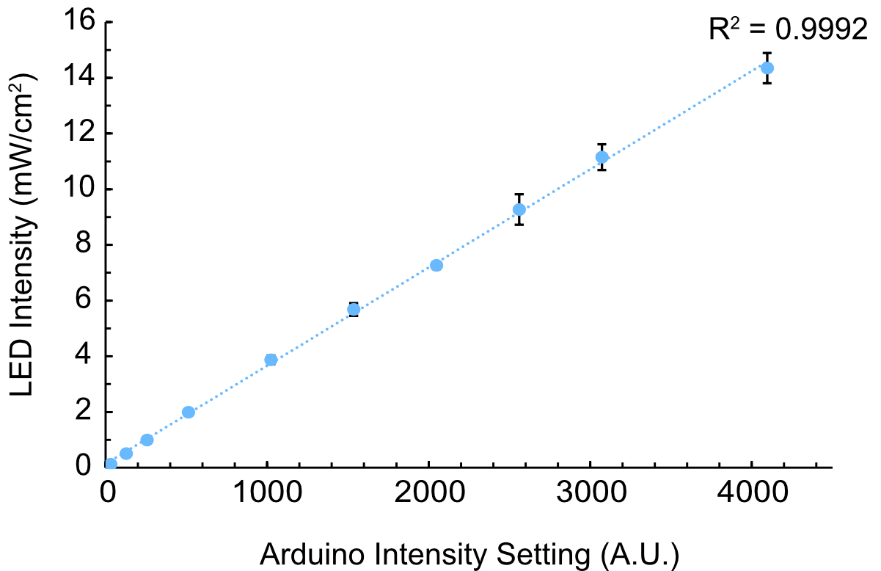


**Figure S3**. Calibration curve of the optoPlate-96. Four LEDs were sampled from the optoPlate-96. Error bars represent standard deviation. Data were fitted with a line and the intercept was fixed to be 0.


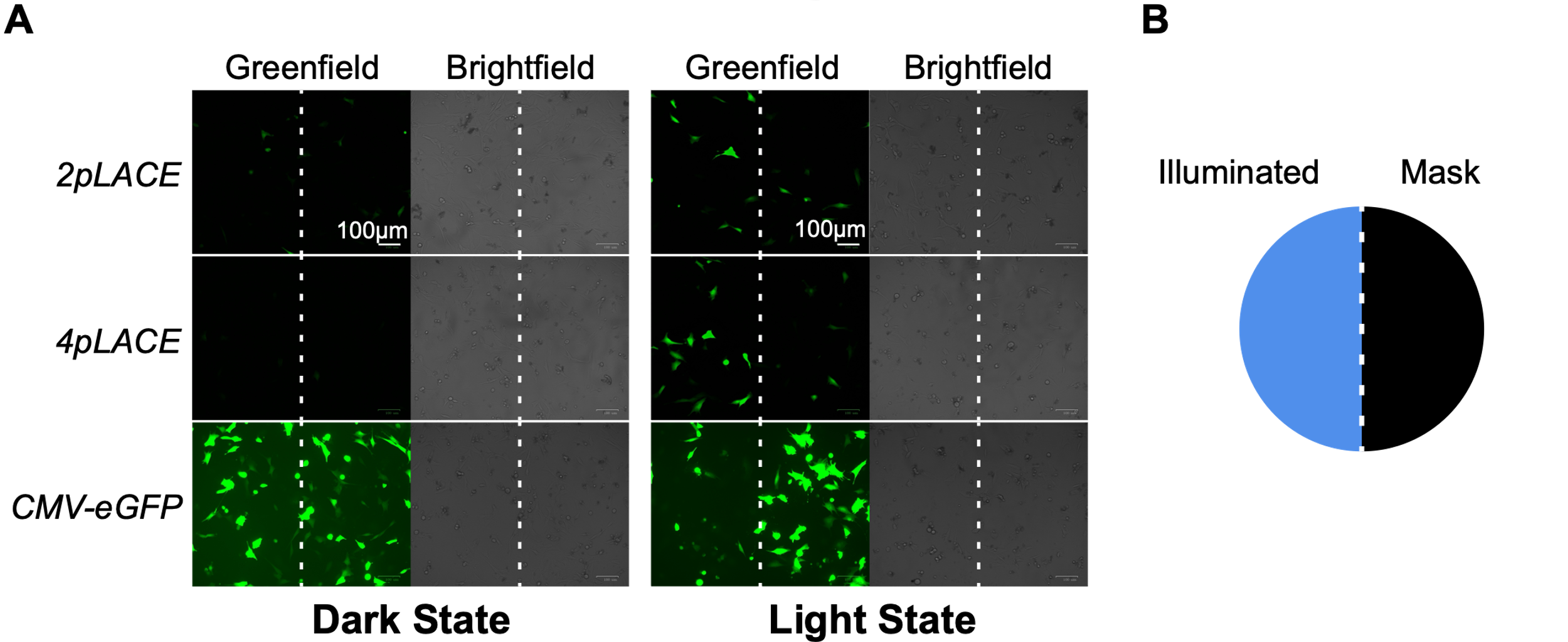


**Figure S4.** Spatial control of eGFP expression with 2pLACE and 4pLACE in C2C12 mouse myoblasts. (A) Images of eGFP expression after 24 hours of illumination where half of the well was blocked, denoted by the dashed white line. CMV-eGFP is the constitutively expressing positive control. (B) Diagram of the electric tape mask on a single well.

**Table S1.** P-values from ANOVA statistics comparing dynamic range of plasmid ratio eGFP expression

|  | **UT** | **1:9** | **2:8** | **3:7** | **4:6** | **5:5** | **6:4** | **7:3** | **8:2** | **9:1** |
| --- | --- | --- | --- | --- | --- | --- | --- | --- | --- | --- |
| **UT** | 1.00 | 4.39E-2 | 1.44E-2 | 3.05E-5 | 5.46E-4 | 4.63E-2 | 2.24E-4 | 1.57E-3 | 1.72E-2 | 5.23E-3 |
| **1:9** | 4.39E-2 | 1.00 | 1.00 | 9.99E-1 | 9.64E-1 | 4.26E-1 | 3.66E-1 | 2.66E-1 | 1.29E-1 | 8.98E-2 |
| **2:8** | 1.44E-2 | 1.00 | 1.00 | 9.14E-1 | 9.42E-1 | 3.37E-1 | 1.78E-1 | 1.03E-1 | 3.67E-2 | 2.50E-2 |
| **3:7** | 3.05E-5 | 9.99E-1 | 9.14E-1 | 1.00 | 7.81E-2 | 2.08E-1 | 1.74E-3 | 3.47E-3 | 1.22E-2 | 3.40E-4 |
| **4:6** | 5.46E-4 | 9.64E-1 | 9.42E-1 | 7.81E-2 | 1.00 | 6.72E-1 | 4.33E-2 | 2.65E-2 | 2.68E-2 | 1.56E-3 |
| **5:5** | 4.63E-2 | 4.26E-1 | 3.37E-1 | 2.08E-1 | 6.72E-1 | 1.00 | 9.99E-1 | 9.51E-1 | 5.33E-1 | 1.62E-1 |
| **6:4** | 2.24E-4 | 3.66E-1 | 1.78E-1 | 1.74E-3 | 4.33E-2 | 9.99E-1 | 1.00 | 9.59E-1 | 4.12E-1 | 7.42E-3 |
| **7:3** | 1.57E-3 | 2.66E-1 | 1.03E-1 | 3.47E-3 | 2.65E-2 | 9.51E-1 | 9.59E-1 | 1.00 | 7.55E-1 | 2.01E-2 |
| **8:2** | 1.72E-2 | 1.29E-1 | 3.67E-2 | 1.22E-2 | 2.68E-2 | 5.33E-1 | 4.12E-1 | 7.55E-1 | 1.00 | 2.37E-1 |
| **9:1** | 5.23E-3 | 8.98E-2 | 2.50E-2 | 3.40E-4 | 1.56E-3 | 1.62E-1 | 7.42E-3 | 2.01E-2 | 2.37E-1 | 1.00 |

**Table S2.** P-values from ANOVA statistics comparing eGFP expression of LED intensity samples (mW/cm^2^)

|  | **0** | **0.12** | **0.50** | **0.99** | **1.99** | **3.87** | **5.68** | **7.26** | **9.27** | **11.15** | **14.35** |
| --- | --- | --- | --- | --- | --- | --- | --- | --- | --- | --- | --- |
| **0** | 1.00 | 1.36E-3 | 1.89E-1 | 8.15E-3 | 6.75E-6 | 1.06E-2 | 6.14E-3 | 3.04E-3 | 3.61E-3 | 3.52E-4 | 3.28E-3 |
| **0.12** | 1.36E-3 | 1.00 | 9.07E-1 | 9.38E-1 | 8.52E-2 | 4.29E-1 | 4.69E-1 | 4.46E-2 | 2.50E-1 | 3.72E-2 | 5.59E-2 |
| **0.50** | 1.89E-1 | 9.07E-1 | 1.00 | 4.48E-1 | 1.37E-1 | 1.39E-1 | 1.56E-1 | 1.25E-2 | 8.53E-2 | 4.28E-2 | 1.61E-2 |
| **0.99** | 8.15E-3 | 9.38E-1 | 4.48E-1 | 1.00 | 9.99E-1 | 9.70E-1 | 9.97E-1 | 2.15E-1 | 9.58E-1 | 8.32E-1 | 2.85E-1 |
| **1.99** | 6.75E-6 | 8.52E-2 | 1.37E-1 | 9.99E-1 | 1.00 | 9.98E-1 | 1.00 | 3.25E-1 | 9.97E-1 | 9.01E-1 | 4.13E-1 |
| **3.87** | 1.06E-2 | 4.29E-1 | 1.39E-1 | 9.70E-1 | 9.98E-1 | 1.00 | 1.00 | 9.09E-1 | 1.00 | 1.00 | 9.62E-1 |
| **5.68** | 6.14E-3 | 4.69E-1 | 1.56E-1 | 9.97E-1 | 1.00 | 1.00 | 1.00 | 6.43E-1 | 1.00 | 1.00 | 7.62E-1 |
| **7.26** | 3.04E-3 | 4.46E-2 | 1.25E-2 | 2.15E-1 | 3.25E-1 | 9.09E-1 | 6.43E-1 | 1.00 | 7.87E-1 | 7.46E-1 | 1.00 |
| **9.27** | 3.61E-3 | 2.50E-1 | 8.53E-2 | 9.58E-1 | 9.97E-1 | 1.00 | 1.00 | 7.87E-1 | 1.00 | 1.00 | 8.86E-1 |
| **11.15** | 3.52E-4 | 3.72E-2 | 4.28E-2 | 8.32E-1 | 9.01E-1 | 1.00 | 1.00 | 7.46E-1 | 1.00 | 1.00 | 8.59E-1 |
| **14.35** | 3.28E-3 | 5.59E-2 | 1.61E-2 | 2.85E-1 | 4.13E-1 | 9.62E-1 | 7.62E-1 | 1.00 | 8.86E-1 | 8.59E-1 | 1.00 |

**Table S3.** P-values from ANOVA statistics comparing conditions of activation time (hours)

|  | **0** | **0.5** | **1** | **2** | **4** | **6** | **12** | **15** | **18** | **21** | **24** |
| --- | --- | --- | --- | --- | --- | --- | --- | --- | --- | --- | --- |
| **0** | 1.00 | 9.83E-1 | 8.50E-1 | 4.35E-1 | 1.57E-2 | 6.17E-4 | 3.55E-7 | 3.15E-4 | 2.29E-3 | 2.52E-5 | 1.20E-5 |
| **0.5** | 9.83E-1 | 1.00 | 1.00 | 7.47E-1 | 4.08E-2 | 2.14E-3 | 4.10E-6 | 5.17E-4 | 2.90E-3 | 6.31E-5 | 2.67E-5 |
| **1** | 8.50E-1 | 1.00 | 1.00 | 9.91E-1 | 6.97E-2 | 2.95E-3 | 4.36E-6 | 5.44E-4 | 3.04E-3 | 6.59E-5 | 2.75E-5 |
| **2** | 4.35E-1 | 7.47E-1 | 9.91E-1 | 1.00 | 1.45E-1 | 8.02E-3 | 2.26E-5 | 7.04E-4 | 3.44E-3 | 1.07E-4 | 4.18E-5 |
| **4** | 1.57E-2 | 4.08E-2 | 6.97E-2 | 1.45E-1 | 1.00 | 2.05E-1 | 9.18E-7 | 5.39E-4 | 4.04E-3 | 3.25E-5 | 1.24E-5 |
| **6** | 6.17E-4 | 2.14E-3 | 2.95E-3 | 8.02E-3 | 2.05E-1 | 1.00 | 4.09E-6 | 8.84E-4 | 6.51E-3 | 4.40E-5 | 1.32E-5 |
| **12** | 3.55E-7 | 4.10E-6 | 4.36E-6 | 2.26E-5 | 9.18E-7 | 4.09E-6 | 1.00 | 1.76E-1 | 2.78E-1 | 8.89E-3 | 2.06E-4 |
| **15** | 3.15E-4 | 5.17E-4 | 5.44E-4 | 7.04E-4 | 5.39E-4 | 8.84E-4 | 1.76E-1 | 1.00 | 1.00 | 9.92E-1 | 9.15E-3 |
| **18** | 2.29E-3 | 2.90E-3 | 3.04E-3 | 3.44E-3 | 4.04E-3 | 6.51E-3 | 2.78E-1 | 1.00 | 1.00 | 1.00 | 1.76E-1 |
| **21** | 2.52E-5 | 6.31E-5 | 6.59E-5 | 1.07E-4 | 3.25E-5 | 4.40E-5 | 8.89E-3 | 9.92E-01 | 1.00 | 1.00 | 1.61E-2 |
| **24** | 1.20E-5 | 2.67E-5 | 2.75E-5 | 4.18E-5 | 1.24E-5 | 1.32E-5 | 2.06E-4 | 9.15E-3 | 1.76E-1 | 1.61E-2 | 1.00 |

**Table S4.** P-values from ANOVA statistics comparing pulse number conditions

|  | **0** | **1** | **4** | **40** | **120** | **480** | **1440** | **2880** | **4320** | **5760** |
| --- | --- | --- | --- | --- | --- | --- | --- | --- | --- | --- |
| **0** | 1.00 | 9.85E-1 | 1.00 | 4.01E-1 | 6.39E-4 | 3.08E-2 | 1.60E-3 | 6.82E-5 | 2.58E-3 | 4.16E-4 |
| **1** | 9.85E-1 | 1.00 | 9.98E-1 | 1.96E-1 | 1.10E-3 | 1.41E-2 | 7.55E-4 | 2.26E-5 | 2.01E-3 | 2.72E-4 |
| **4** | 1.00 | 9.98E-1 | 1.00 | 2.01E-1 | 4.34E-4 | 2.97E-2 | 1.80E-3 | 9.18E-5 | 2.62E-3 | 4.48E-4 |
| **40** | 4.01E-1 | 1.96E-1 | 2.01E-1 | 1.00 | 2.36E-2 | 9.04E-2 | 2.20E-3 | 6.51E-5 | 3.09E-3 | 4.60E-4 |
| **120** | 6.39E-4 | 1.10E-3 | 4.34E-4 | 2.36E-2 | 1.00 | 7.89E-1 | 6.91E-3 | 1.31E-4 | 4.98E-3 | 7.32E-4 |
| **480** | 3.08E-2 | 1.41E-2 | 2.97E-2 | 9.04E-2 | 7.89E-1 | 1.00 | 1.68E-2 | 2.96E-5 | 4.34E-3 | 2.87E-4 |
| **1440** | 1.60E-3 | 7.55E-4 | 1.80E-3 | 2.20E-3 | 6.91E-3 | 1.68E-2 | 1.00 | 1.00E-2 | 4.86E-2 | 3.32E-3 |
| **2880** | 6.82E-5 | 2.26E-5 | 9.18E-5 | 6.51E-5 | 1.31E-4 | 2.96E-5 | 1.00E-2 | 1.00 | 9.55E-1 | 3.36E-1 |
| **4320** | 2.58E-3 | 2.01E-3 | 2.62E-3 | 3.09E-3 | 4.98E-3 | 4.34E-3 | 4.86E-2 | 9.55E-1 | 1.00 | 9.99E-1 |
| **5760** | 4.16E-4 | 2.72E-4 | 4.48E-4 | 4.60E-4 | 7.32E-4 | 2.87E-4 | 3.32E-3 | 3.36E-1 | 9.99E-1 | 1.00 |
